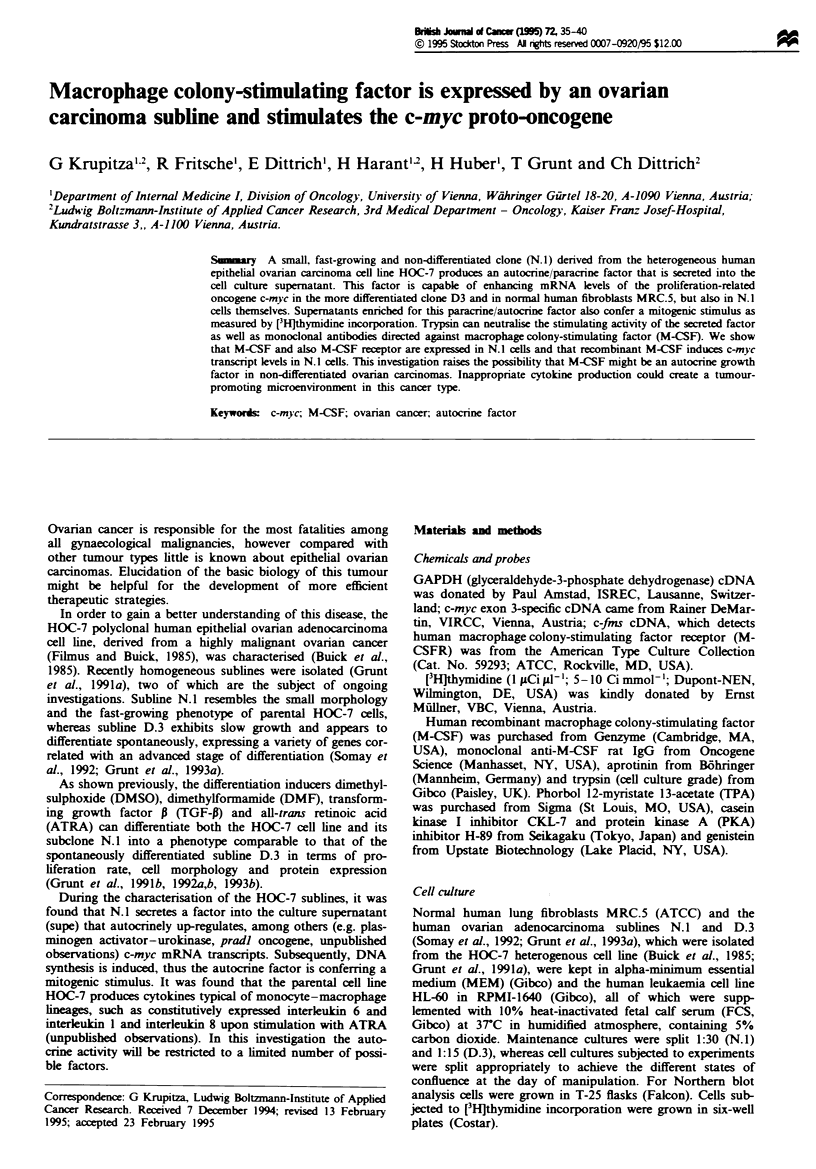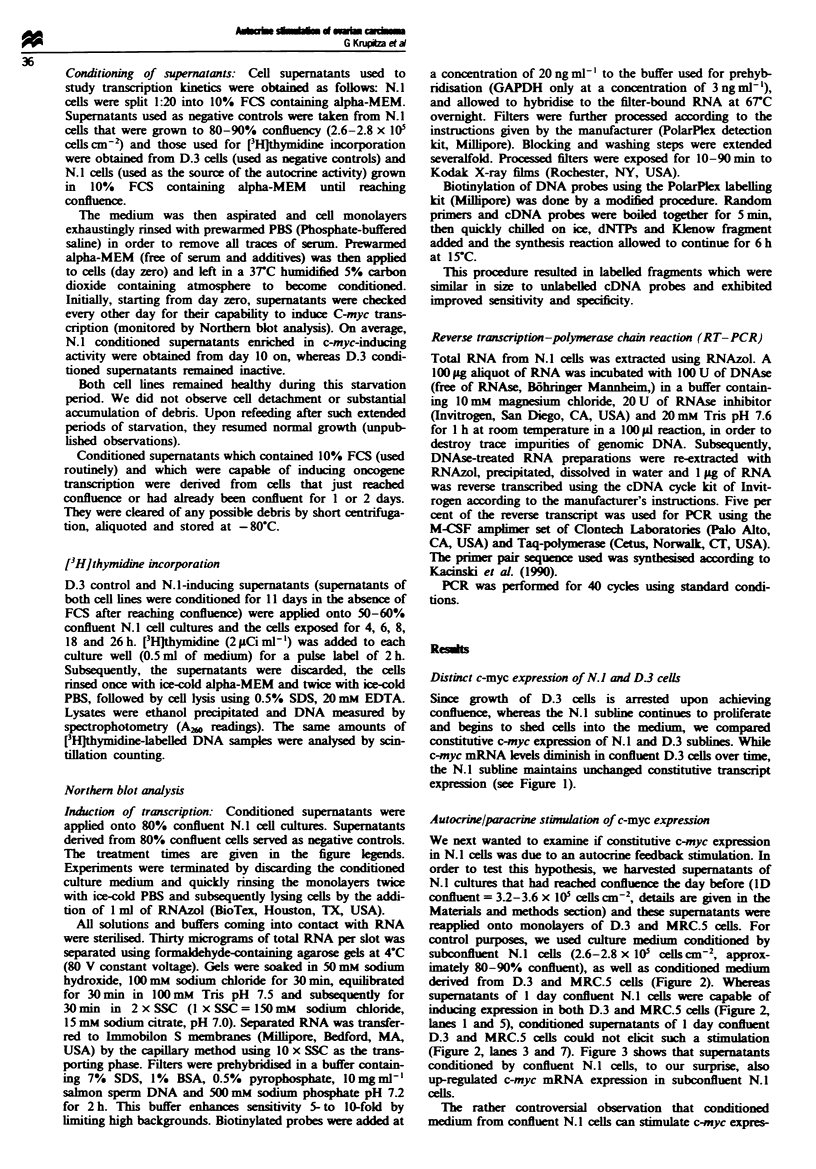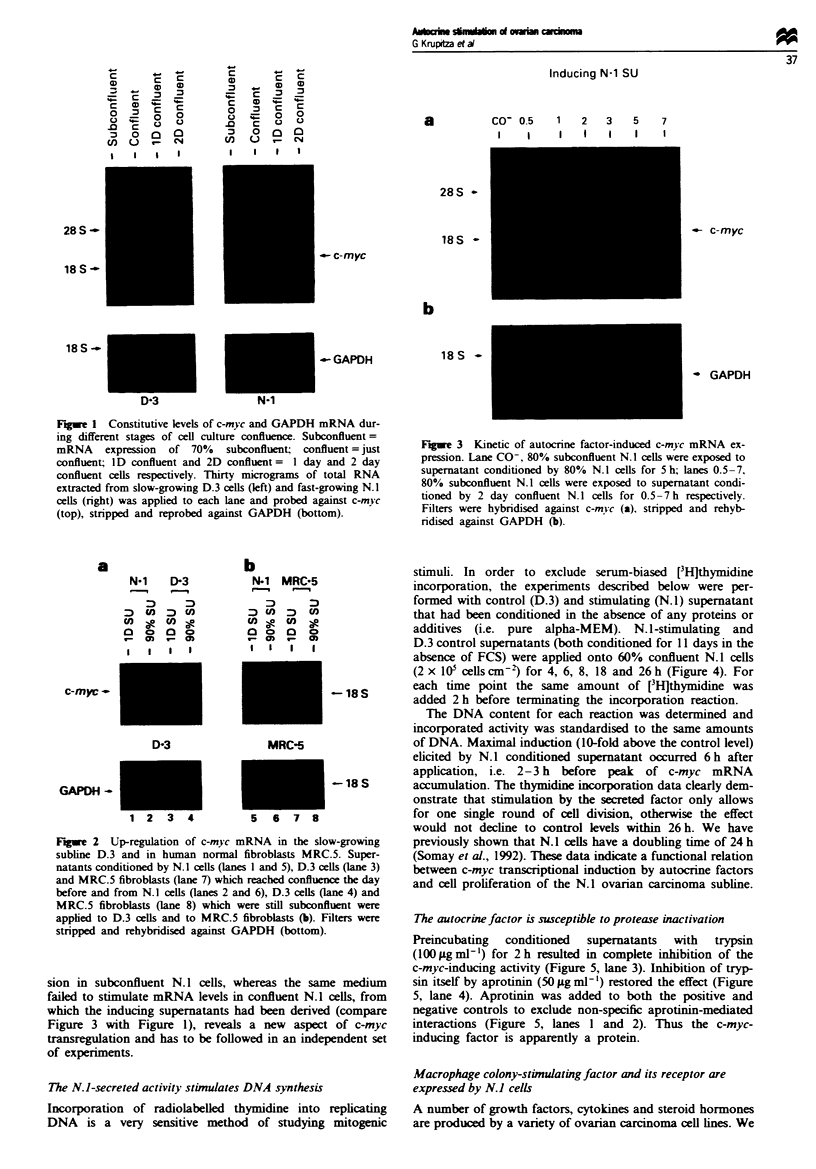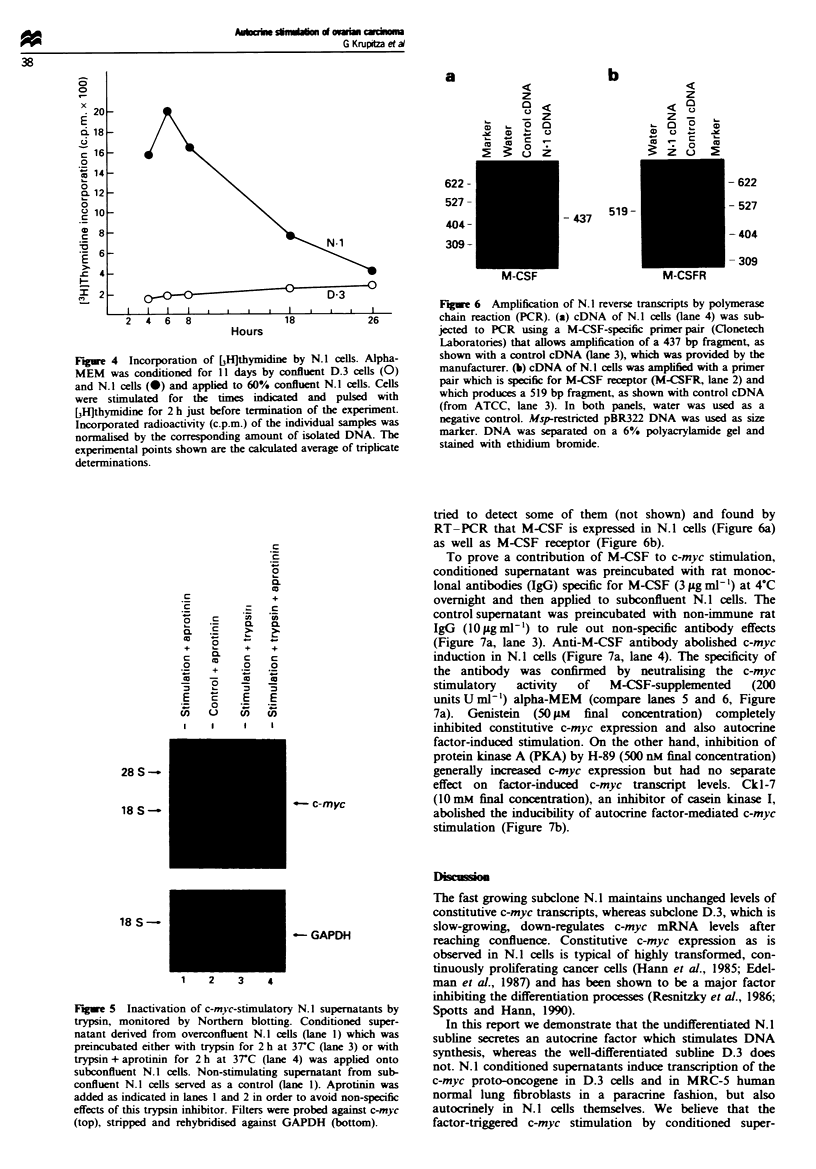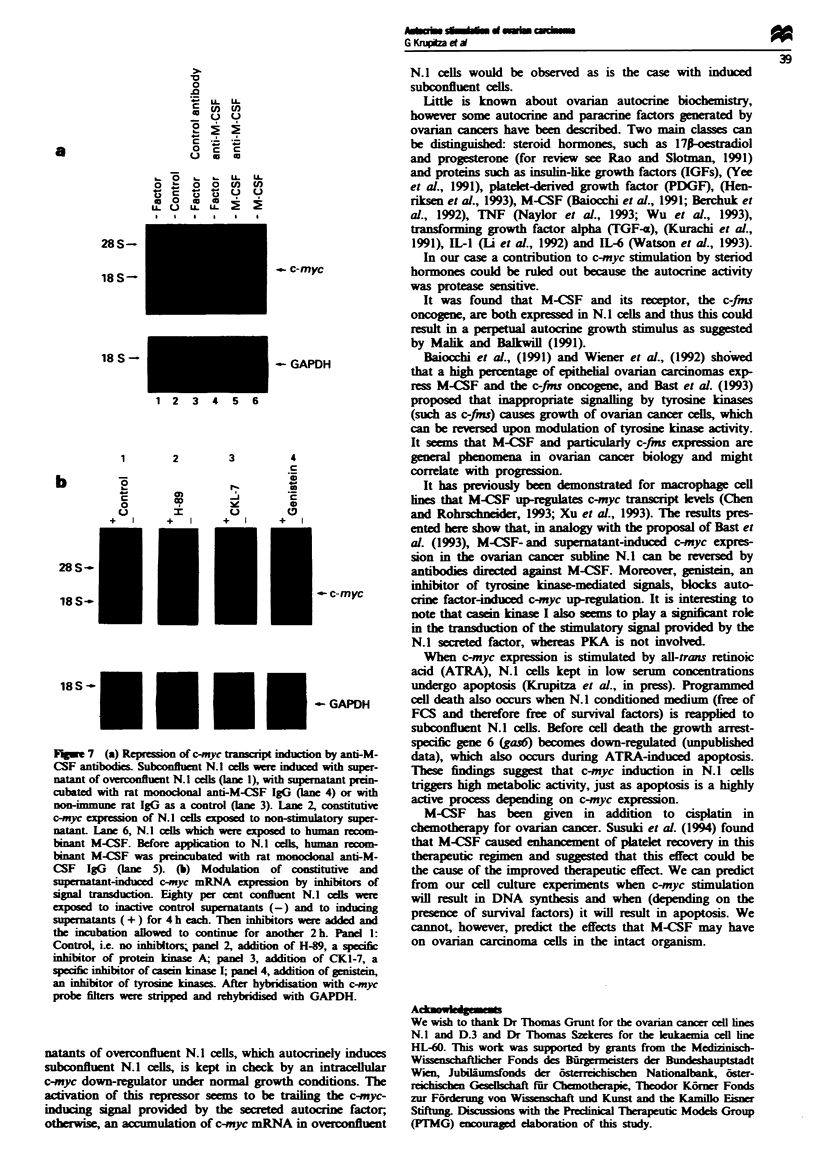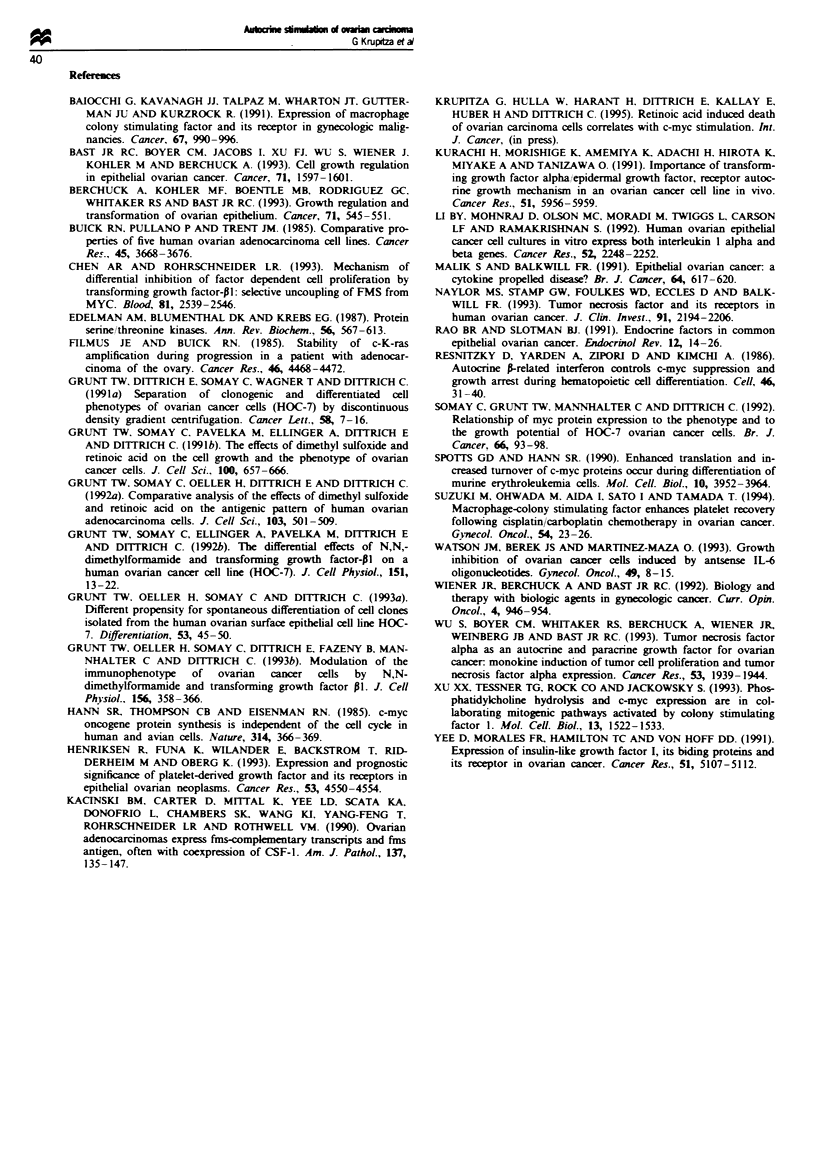# Macrophage colony-stimulating factor is expressed by an ovarian carcinoma subline and stimulates the c-myc proto-oncogene.

**DOI:** 10.1038/bjc.1995.273

**Published:** 1995-07

**Authors:** G. Krupitza, R. Fritsche, E. Dittrich, H. Harant, H. Huber, T. Grunt, C. Dittrich

**Affiliations:** Department of Internal Medicine I, University of Vienna, Austria.

## Abstract

**Images:**


British Journal of Cancer (1995) 72, 35-40

© 1995 Stockton Press All rights reserved 0007-0920/95 $12.00

### Macrophage colony-stimulating factor is expressed by an ovarian carcinoma subline and stimulates the c-*myc* proto-oncogene

G Krupitza[1,2], R Fritsche[1], E Dittrich[1], H Harant[1,2], H Huber[1], T Grunt and Ch Dittrich[2]

[1]*Department of Internal Medicine I, Division of Oncology, University of Vienna, Währinger Gürtel 18-20, A-1090 Vienna, Austria;* [2]*Ludwig Boltzmann-Institute of Applied Cancer Research, 3rd Medical Department – Oncology, Kaiser Franz Josef-Hospital, Kundratstrasse 3,, A-1100 Vienna, Austria.*

**Summary** A small, fast-growing and non-differentiated clone (N.1) derived from the heterogeneous human epithelial ovarian carcinoma cell line HOC-7 produces an autocrine/paracrine factor that is secreted into the cell culture supernatant. This factor is capable of enhancing mRNA levels of the proliferation-related oncogene c-*myc* in the more differentiated clone D3 and in normal human fibroblasts MRC.5, but also in N.1 cells themselves. Supernatants enriched for this paracrine/autocrine factor also confer a mitogenic stimulus as measured by [$^3$H]thymidine incorporation. Trypsin can neutralise the stimulating activity of the secreted factor as well as monoclonal antibodies directed against macrophage colony-stimulating factor (M-CSF). We show that M-CSF and also M-CSF receptor are expressed in N.1 cells and that recombinant M-CSF induces c-*myc* transcript levels in N.1 cells. This investigation raises the possibility that M-CSF might be an autocrine growth factor in non-differentiated ovarian carcinomas. Inappropriate cytokine production could create a tumour-promoting microenvironment in this cancer type.

**Keywords:** c-*myc*; M-CSF; ovarian cancer; autocrine factor

Ovarian cancer is responsible for the most fatalities among all gynaecological malignancies, however compared with other tumour types little is known about epithelial ovarian carcinomas. Elucidation of the basic biology of this tumour might be helpful for the development of more efficient therapeutic strategies.

In order to gain a better understanding of this disease, the HOC-7 polyclonal human epithelial ovarian adenocarcinoma cell line, derived from a highly malignant ovarian cancer (Filmus and Buick, 1985), was characterised (Buick *et al.*, 1985). Recently homogeneous sublines were isolated (Grunt *et al.*, 1991*a*), two of which are the subject of ongoing investigations. Subline N.1 resembles the small morphology and the fast-growing phenotype of parental HOC-7 cells, whereas subline D.3 exhibits slow growth and appears to differentiate spontaneously, expressing a variety of genes correlated with an advanced stage of differentiation (Somay *et al.*, 1992; Grunt *et al.*, 1993*a*).

As shown previously, the differentiation inducers dimethylsulphoxide (DMSO), dimethylformamide (DMF), transforming growth factor $\beta$ (TGF-$\beta$) and all-*trans* retinoic acid (ATRA) can differentiate both the HOC-7 cell line and its subclone N.1 into a phenotype comparable to that of the spontaneously differentiated subline D.3 in terms of proliferation rate, cell morphology and protein expression (Grunt *et al.*, 1991*b*, 1992*a*,*b*, 1993*b*).

During the characterisation of the HOC-7 sublines, it was found that N.1 secretes a factor into the culture supernatant (supe) that autocrinely up-regulates, among others (e.g. plasminogen activator-urokinase, *pradl* oncogene, unpublished observations) c-*myc* mRNA transcripts. Subsequently, DNA synthesis is induced, thus the autocrine factor is conferring a mitogenic stimulus. It was found that the parental cell line HOC-7 produces cytokines typical of monocyte-macrophage lineages, such as constitutively expressed interleukin 6 and interleukin 1 and interleukin 8 upon stimulation with ATRA (unpublished observations). In this investigation the autocrine activity will be restricted to a limited number of possible factors.

Correspondence: G Krupitza, Ludwig Boltzmann-Institute of Applied Cancer Research. Received 7 December 1994; revised 13 February 1995; accepted 23 February 1995

### Materials and methods

### Chemicals and probes

GAPDH (glyceraldehyde-3-phosphate dehydrogenase) cDNA was donated by Paul Amstad, ISREC, Lausanne, Switzerland; c-*myc* exon 3-specific cDNA came from Rainer DeMartin, VIRCC, Vienna, Austria; c-*fms* cDNA, which detects human macrophage colony-stimulating factor receptor (M-CSFR) was from the American Type Culture Collection (Cat. No. 59293; ATCC, Rockville, MD, USA).

[$^3$H]thymidine (1 $\mu$Ci $\mu$l$^{-1}$; 5-10 Ci mmol$^{-1}$; Dupont-NEN, Wilmington, DE, USA) was kindly donated by Ernst Müllner, VBC, Vienna, Austria.

Human recombinant macrophage colony-stimulating factor (M-CSF) was purchased from Genzyme (Cambridge, MA, USA), monoclonal anti-M-CSF rat IgG from Oncogene Science (Manhasset, NY, USA), aprotinin from Böhringer (Mannheim, Germany) and trypsin (cell culture grade) from Gibco (Paisley, UK). Phorbol 12-myristate 13-acetate (TPA) was purchased from Sigma (St Louis, MO, USA), casein kinase I inhibitor CKL-7 and protein kinase A (PKA) inhibitor H-89 from Seikagaku (Tokyo, Japan) and genistein from Upstate Biotechnology (Lake Placid, NY, USA).

### Cell culture

Normal human lung fibroblasts MRC.5 (ATCC) and the human ovarian adenocarcinoma sublines N.1 and D.3 (Somay *et al.*, 1992; Grunt *et al.*, 1993*a*), which were isolated from the HOC-7 heterogenous cell line (Buick *et al.*, 1985; Grunt *et al.*, 1991*a*), were kept in alpha-minimum essential medium (MEM) (Gibco) and the human leukaemia cell line HL-60 in RPMI-1640 (Gibco), all of which were supplemented with 10% heat-inactivated fetal calf serum (FCS, Gibco) at 37°C in humidified atmosphere, containing 5% carbon dioxide. Maintenance cultures were split 1:30 (N.1) and 1:15 (D.3), whereas cell cultures subjected to experiments were split appropriately to achieve the different states of confluence at the day of manipulation. For Northern blot analysis cells were grown in T-25 flasks (Falcon). Cells subjected to [$^3$H]thymidine incorporation were grown in six-well plates (Costar).

*Conditioning of supernatants:* Cell supernatants used to study transcription kinetics were obtained as follows: N.1 cells were split 1:20 into 10% FCS containing alpha-MEM. Supernatants used as negative controls were taken from N.1 cells that were grown to 80–90% confluency ($2.6-2.8 \times 10^5$ cells $cm^{-2}$) and those used for [$^3$H]thymidine incorporation were obtained from D.3 cells (used as negative controls) and N.1 cells (used as the source of the autocrine activity) grown in 10% FCS containing alpha-MEM until reaching confluence.

The medium was then aspirated and cell monolayers exhaustingly rinsed with prewarmed PBS (Phosphate-buffered saline) in order to remove all traces of serum. Prewarmed alpha-MEM (free of serum and additives) was then applied to cells (day zero) and left in a 37°C humidified 5% carbon dioxide containing atmosphere to become conditioned. Initially, starting from day zero, supernatants were checked every other day for their capability to induce *C-myc* transcription (monitored by Northern blot analysis). On average, N.1 conditioned supernatants enriched in *c-myc*-inducing activity were obtained from day 10 on, whereas D.3 conditioned supernatants remained inactive.

Both cell lines remained healthy during this starvation period. We did not observe cell detachment or substantial accumulation of debris. Upon refeeding after such extended periods of starvation, they resumed normal growth (unpublished observations).

Conditioned supernatants which contained 10% FCS (used routinely) and which were capable of inducing oncogene transcription were derived from cells that just reached confluence or had already been confluent for 1 or 2 days. They were cleared of any possible debris by short centrifugation, aliquoted and stored at −80°C.

### *[$^3$H]thymidine incorporation*

D.3 control and N.1-inducing supernatants (supernatants of both cell lines were conditioned for 11 days in the absence of FCS after reaching confluence) were applied onto 50–60% confluent N.1 cell cultures and the cells exposed for 4, 6, 8, 18 and 26 h. [$^3$H]thymidine (2 $\mu$Ci $ml^{-1}$) was added to each culture well (0.5 ml of medium) for a pulse label of 2 h. Subsequently, the supernatants were discarded, the cells rinsed once with ice-cold alpha-MEM and twice with ice-cold PBS, followed by cell lysis using 0.5% SDS, 20 mM EDTA. Lysates were ethanol precipitated and DNA measured by spectrophotometry ($A_{260}$ readings). The same amounts of [$^3$H]thymidine-labelled DNA samples were analysed by scintillation counting.

### *Northern blot analysis*

*Induction of transcription:* Conditioned supernatants were applied onto 80% confluent N.1 cell cultures. Supernatants derived from 80% confluent cells served as negative controls. The treatment times are given in the figure legends. Experiments were terminated by discarding the conditioned culture medium and quickly rinsing the monolayers twice with ice-cold PBS and subsequently lysing cells by the addition of 1 ml of RNAzol (BioTex, Houston, TX, USA).

All solutions and buffers coming into contact with RNA were sterilised. Thirty micrograms of total RNA per slot was separated using formaldehyde-containing agarose gels at 4°C (80 V constant voltage). Gels were soaked in 50 mM sodium hydroxide, 100 mM sodium chloride for 30 min, equilibrated for 30 min in 100 mM Tris pH 7.5 and subsequently for 30 min in 2 × SSC (1 × SSC = 150 mM sodium chloride, 15 mM sodium citrate, pH 7.0). Separated RNA was transferred to Immobilon S membranes (Millipore, Bedford, MA, USA) by the capillary method using 10 × SSC as the transporting phase. Filters were prehybridised in a buffer containing 7% SDS, 1% BSA, 0.5% pyrophosphate, 10 mg $ml^{-1}$ salmon sperm DNA and 500 mM sodium phosphate pH 7.2 for 2 h. This buffer enhances sensitivity 5- to 10-fold by limiting high backgrounds. Biotinylated probes were added at a concentration of 20 ng $ml^{-1}$ to the buffer used for prehybridisation (GAPDH only at a concentration of 3 ng $ml^{-1}$), and allowed to hybridise to the filter-bound RNA at 67°C overnight. Filters were further processed according to the instructions given by the manufacturer (PolarPlex detection kit, Millipore). Blocking and washing steps were extended severalfold. Processed filters were exposed for 10–90 min to Kodak X-ray films (Rochester, NY, USA).

Biotinylation of DNA probes using the PolarPlex labelling kit (Millipore) was done by a modified procedure. Random primers and cDNA probes were boiled together for 5 min, then quickly chilled on ice, dNTPs and Klenow fragment added and the synthesis reaction allowed to continue for 6 h at 15°C.

This procedure resulted in labelled fragments which were similar in size to unlabelled cDNA probes and exhibited improved sensitivity and specificity.

### *Reverse transcription–polymerase chain reaction (RT–PCR)*

Total RNA from N.1 cells was extracted using RNAzol. A 100 µg aliquot of RNA was incubated with 100 U of DNAse (free of RNAse, Böhringer Mannheim,) in a buffer containing 10 mM magnesium chloride, 20 U of RNAse inhibitor (Invitrogen, San Diego, CA, USA) and 20 mM Tris pH 7.6 for 1 h at room temperature in a 100 µl reaction, in order to destroy trace impurities of genomic DNA. Subsequently, DNAse-treated RNA preparations were re-extracted with RNAzol, precipitated, dissolved in water and 1 µg of RNA was reverse transcribed using the cDNA cycle kit of Invitrogen according to the manufacturer's instructions. Five per cent of the reverse transcript was used for PCR using the M-CSF amplimer set of Clontech Laboratories (Palo Alto, CA, USA) and Taq-polymerase (Cetus, Norwalk, CT, USA). The primer pair sequence used was synthesised according to Kacinski *et al.* (1990).

PCR was performed for 40 cycles using standard conditions.

### Results

### *Distinct* c-myc *expression of N.1 and D.3 cells*

Since growth of D.3 cells is arrested upon achieving confluence, whereas the N.1 subline continues to proliferate and begins to shed cells into the medium, we compared constitutive *c-myc* expression of N.1 and D.3 sublines. While *c-myc* mRNA levels diminish in confluent D.3 cells over time, the N.1 subline maintains unchanged constitutive transcript expression (see Figure 1).

### *Autocrine/paracrine stimulation of* c-myc *expression*

We next wanted to examine if constitutive *c-myc* expression in N.1 cells was due to an autocrine feedback stimulation. In order to test this hypothesis, we harvested supernatants of N.1 cultures that had reached confluence the day before (1D confluent = $3.2-3.6 \times 10^5$ cells $cm^{-2}$, details are given in the Materials and methods section) and these supernatants were reapplied onto monolayers of D.3 and MRC.5 cells. For control purposes, we used culture medium conditioned by subconfluent N.1 cells ($2.6-2.8 \times 10^5$ cells $cm^{-2}$, approximately 80–90% confluent), as well as conditioned medium derived from D.3 and MRC.5 cells (Figure 2). Whereas supernatants of 1 day confluent N.1 cells were capable of inducing expression in both D.3 and MRC.5 cells (Figure 2, lanes 1 and 5), conditioned supernatants of 1 day confluent D.3 and MRC.5 cells could not elicit such a stimulation (Figure 2, lanes 3 and 7). Figure 3 shows that supernatants conditioned by confluent N.1 cells, to our surprise, also up-regulated *c-myc* mRNA expression in subconfluent N.1 cells.

The rather controversial observation that conditioned medium from confluent N.1 cells can stimulate *c-myc* expres-

**Figure 1** Constitutive levels of c-*myc* and GAPDH mRNA during different stages of cell culture confluence. Subconfluent = mRNA expression of 70% subconfluent; confluent = just confluent; 1D confluent and 2D confluent = 1 day and 2 day confluent cells respectively. Thirty micrograms of total RNA extracted from slow-growing D.3 cells (left) and fast-growing N.1 cells (right) was applied to each lane and probed against c-*myc* (top), stripped and reprobed against GAPDH (bottom).

**Figure 3** Kinetic of autocrine factor-induced c-*myc* mRNA expression. Lane $CO^-$, 80% subconfluent N.1 cells were exposed to supernatant conditioned by 80% N.1 cells for 5 h; lanes 0.5–7, 80% subconfluent N.1 cells were exposed to supernatant conditioned by 2 day confluent N.1 cells for 0.5–7 h respectively. Filters were hybridised against c-*myc* (**a**), stripped and rehybridised against GAPDH (**b**).

**Figure 2** Up-regulation of c-*myc* mRNA in the slow-growing subline D.3 and in human normal fibroblasts MRC.5. Supernatants conditioned by N.1 cells (lanes 1 and 5), D.3 cells (lane 3) and MRC.5 fibroblasts (lane 7) which reached confluence the day before and from N.1 cells (lanes 2 and 6), D.3 cells (lane 4) and MRC.5 fibroblasts (lane 8) which were still subconfluent were applied to D.3 cells and to MRC.5 fibroblasts (**b**). Filters were stripped and rehybridised against GAPDH (bottom).

sion in subconfluent N.1 cells, whereas the same medium failed to stimulate mRNA levels in confluent N.1 cells, from which the inducing supernatants had been derived (compare Figure 3 with Figure 1), reveals a new aspect of c-*myc* transregulation and has to be followed in an independent set of experiments.

### *The N.1-secreted activity stimulates DNA synthesis*

Incorporation of radiolabelled thymidine into replicating DNA is a very sensitive method of studying mitogenic stimuli. In order to exclude serum-biased [$^3$H]thymidine incorporation, the experiments described below were performed with control (D.3) and stimulating (N.1) supernatant that had been conditioned in the absence of any proteins or additives (i.e. pure alpha-MEM). N.1-stimulating and D.3 control supernatants (both conditioned for 11 days in the absence of FCS) were applied onto 60% confluent N.1 cells ($2 \times 10^5$ cells $cm^{-2}$) for 4, 6, 8, 18 and 26 h (Figure 4). For each time point the same amount of [$^3$H]thymidine was added 2 h before terminating the incorporation reaction.

The DNA content for each reaction was determined and incorporated activity was standardised to the same amounts of DNA. Maximal induction (10-fold above the control level) elicited by N.1 conditioned supernatant occurred 6 h after application, i.e. 2–3 h before peak of c-*myc* mRNA accumulation. The thymidine incorporation data clearly demonstrate that stimulation by the secreted factor only allows for one single round of cell division, otherwise the effect would not decline to control levels within 26 h. We have previously shown that N.1 cells have a doubling time of 24 h (Somay *et al.*, 1992). These data indicate a functional relation between c-*myc* transcriptional induction by autocrine factors and cell proliferation of the N.1 ovarian carcinoma subline.

### *The autocrine factor is susceptible to protease inactivation*

Preincubating conditioned supernatants with trypsin (100 $\mu g\ ml^{-1}$) for 2 h resulted in complete inhibition of the c-*myc*-inducing activity (Figure 5, lane 3). Inhibition of trypsin itself by aprotinin (50 $\mu g\ ml^{-1}$) restored the effect (Figure 5, lane 4). Aprotinin was added to both the positive and negative controls to exclude non-specific aprotinin-mediated interactions (Figure 5, lanes 1 and 2). Thus the c-*myc*-inducing factor is apparently a protein.

### *Macrophage colony-stimulating factor and its receptor are expressed by N.1 cells*

A number of growth factors, cytokines and steroid hormones are produced by a variety of ovarian carcinoma cell lines. We

**Figure 4** Incorporation of [3H]thymidine by N.1 cells. Alpha-MEM was conditioned for 11 days by confluent D.3 cells (○) and N.1 cells (●) and applied to 60% confluent N.1 cells. Cells were stimulated for the times indicated and pulsed with [3H]thymidine for 2 h just before termination of the experiment. Incorporated radioactivity (c.p.m.) of the individual samples was normalised by the corresponding amount of isolated DNA. The experimental points shown are the calculated average of triplicate determinations.

**Figure 6** Amplification of N.1 reverse transcripts by polymerase chain reaction (PCR). (**a**) cDNA of N.1 cells (lane 4) was subjected to PCR using a M-CSF-specific primer pair (Clonetech Laboratories) that allows amplification of a 437 bp fragment, as shown with a control cDNA (lane 3), which was provided by the manufacturer. (**b**) cDNA of N.1 cells was amplified with a primer pair which is specific for M-CSF receptor (M-CSFR, lane 2) and which produces a 519 bp fragment, as shown with control cDNA (from ATCC, lane 3). In both panels, water was used as a negative control. *Msp*-restricted pBR322 DNA was used as size marker. DNA was separated on a 6% polyacrylamide gel and stained with ethidium bromide.

**Figure 5** Inactivation of c-*myc*-stimulatory N.1 supernatants by trypsin, monitored by Northern blotting. Conditioned supernatant derived from overconfluent N.1 cells (lane 1) which was preincubated either with trypsin for 2 h at 37°C (lane 3) or with trypsin + aprotinin for 2 h at 37°C (lane 4) was applied onto subconfluent N.1 cells. Non-stimulating supernatant from subconfluent N.1 cells served as a control (lane 1). Aprotinin was added as indicated in lanes 1 and 2 in order to avoid non-specific effects of this trypsin inhibitor. Filters were probed against c-*myc* (top), stripped and rehybridised against GAPDH (bottom).

tried to detect some of them (not shown) and found by RT–PCR that M-CSF is expressed in N.1 cells (Figure 6a) as well as M-CSF receptor (Figure 6b).

To prove a contribution of M-CSF to c-*myc* stimulation, conditioned supernatant was preincubated with rat monoclonal antibodies (IgG) specific for M-CSF (3 $\mu g\ ml^{-1}$) at 4°C overnight and then applied to subconfluent N.1 cells. The control supernatant was preincubated with non-immune rat IgG (10 $\mu g\ ml^{-1}$) to rule out non-specific antibody effects (Figure 7a, lane 3). Anti-M-CSF antibody abolished c-*myc* induction in N.1 cells (Figure 7a, lane 4). The specificity of the antibody was confirmed by neutralising the c-*myc* stimulatory activity of M-CSF-supplemented (200 units U $ml^{-1}$) alpha-MEM (compare lanes 5 and 6, Figure 7a). Genistein (50 μM final concentration) completely inhibited constitutive c-*myc* expression and also autocrine factor-induced stimulation. On the other hand, inhibition of protein kinase A (PKA) by H-89 (500 nM final concentration) generally increased c-*myc* expression but had no separate effect on factor-induced c-*myc* transcript levels. Ck1-7 (10 mM final concentration), an inhibitor of casein kinase I, abolished the inducibility of autocrine factor-mediated c-*myc* stimulation (Figure 7b).

### Discussion

The fast growing subclone N.1 maintains unchanged levels of constitutive c-*myc* transcripts, whereas subclone D.3, which is slow-growing, down-regulates c-*myc* mRNA levels after reaching confluence. Constitutive c-*myc* expression as is observed in N.1 cells is typical of highly transformed, continuously proliferating cancer cells (Hann *et al*., 1985; Edelman *et al*., 1987) and has been shown to be a major factor inhibiting the differentiation processes (Resnitzky *et al*., 1986; Spotts and Hann, 1990).

In this report we demonstrate that the undifferentiated N.1 subline secretes an autocrine factor which stimulates DNA synthesis, whereas the well-differentiated subline D.3 does not. N.1 conditioned supernatants induce transcription of the c-*myc* proto-oncogene in D.3 cells and in MRC-5 human normal lung fibroblasts in a paracrine fashion, but also autocrinely in N.1 cells themselves. We believe that the factor-triggered c-*myc* stimulation by conditioned super-

**Figure 7** (**a**) Repression of c-*myc* transcript induction by anti-M-CSF antibodies. Subconfluent N.1 cells were induced with supernatant of overconfluent N.1 cells (lane 1), with supernatant preincubated with rat monoclonal anti-M-CSF IgG (lane 4) or with non-immune rat IgG as a control (lane 3). Lane 2, constitutive c-*myc* expression of N.1 cells exposed to non-stimulatory supernatant. Lane 6, N.1 cells which were exposed to human recombinant M-CSF. Before application to N.1 cells, human recombinant M-CSF was preincubated with rat monoclonal anti-M-CSF IgG (lane 5). (**b**) Modulation of constitutive and supernatant-induced c-*myc* mRNA expression by inhibitors of signal transduction. Eighty per cent confluent N.1 cells were exposed to inactive control supernatants (−) and to inducing supernatants (+) for 4 h each. Then inhibitors were added and the incubation allowed to continue for another 2 h. Panel 1: Control, i.e. no inhibitors; panel 2, addition of H-89, a specific inhibitor of protein kinase A; panel 3, addition of CK1-7, a specific inhibitor of casein kinase I; panel 4, addition of genistein, an inhibitor of tyrosine kinases. After hybridisation with c-*myc* probe filters were stripped and rehybridised with GAPDH.

natants of overconfluent N.1 cells, which autocrinely induces subconfluent N.1 cells, is kept in check by an intracellular c-*myc* down-regulator under normal growth conditions. The activation of this repressor seems to be trailing the c-*myc*-inducing signal provided by the secreted autocrine factor; otherwise, an accumulation of c-*myc* mRNA in overconfluent N.1 cells would be observed as is the case with induced subconfluent cells.

Little is known about ovarian autocrine biochemistry, however some autocrine and paracrine factors generated by ovarian cancers have been described. Two main classes can be distinguished: steroid hormones, such as 17$\beta$-oestradiol and progesterone (for review see Rao and Slotman, 1991) and proteins such as insulin-like growth factors (IGFs), (Yee *et al*., 1991), platelet-derived growth factor (PDGF), (Henriksen *et al*., 1993), M-CSF (Baiocchi *et al*., 1991; Berchuk *et al*., 1992), TNF (Naylor *et al*., 1993; Wu *et al*., 1993), transforming growth factor alpha (TGF-$\alpha$), (Kurachi *et al*., 1991), IL-1 (Li *et al*., 1992) and IL-6 (Watson *et al*., 1993).

In our case a contribution to c-*myc* stimulation by steriod hormones could be ruled out because the autocrine activity was protease sensitive.

It was found that M-CSF and its receptor, the c-*fms* oncogene, are both expressed in N.1 cells and thus this could result in a perpetual autocrine growth stimulus as suggested by Malik and Balkwill (1991).

Baiocchi *et al*., (1991) and Wiener *et al*., (1992) showed that a high percentage of epithelial ovarian carcinomas express M-CSF and the c-*fms* oncogene, and Bast *et al*. (1993) proposed that inappropriate signalling by tyrosine kinases (such as c-*fms*) causes growth of ovarian cancer cells, which can be reversed upon modulation of tyrosine kinase activity. It seems that M-CSF and particularly c-*fms* expression are general phenomena in ovarian cancer biology and might correlate with progression.

It has previously been demonstrated for macrophage cell lines that M-CSF up-regulates c-*myc* transcript levels (Chen and Rohrschneider, 1993; Xu *et al*., 1993). The results presented here show that, in analogy with the proposal of Bast *et al*. (1993), M-CSF- and supernatant-induced c-*myc* expression in the ovarian cancer subline N.1 can be reversed by antibodies directed against M-CSF. Moreover, genistein, an inhibitor of tyrosine kinase-mediated signals, blocks autocrine factor-induced c-*myc* up-regulation. It is interesting to note that casein kinase I also seems to play a significant role in the transduction of the stimulatory signal provided by the N.1 secreted factor, whereas PKA is not involved.

When c-*myc* expression is stimulated by all-*trans* retinoic acid (ATRA), N.1 cells kept in low serum concentrations undergo apoptosis (Krupitza *et al*., in press). Programmed cell death also occurs when N.1 conditioned medium (free of FCS and therefore free of survival factors) is reapplied to subconfluent N.1 cells. Before cell death the growth arrest-specific gene 6 (*gas6*) becomes down-regulated (unpublished data), which also occurs during ATRA-induced apoptosis. These findings suggest that c-*myc* induction in N.1 cells triggers high metabolic activity, just as apoptosis is a highly active process depending on c-*myc* expression.

M-CSF has been given in addition to cisplatin in chemotherapy for ovarian cancer. Susuki *et al*. (1994) found that M-CSF caused enhancement of platelet recovery in this therapeutic regimen and suggested that this effect could be the cause of the improved therapeutic effect. We can predict from our cell culture experiments when c-*myc* stimulation will result in DNA synthesis and when (depending on the presence of survival factors) it will result in apoptosis. We cannot, however, predict the effects that M-CSF may have on ovarian carcinoma cells in the intact organism.

**Acknowledgements**

We wish to thank Dr Thomas Grunt for the ovarian cancer cell lines N.1 and D.3 and Dr Thomas Szekeres for the leukaemia cell line HL-60. This work was supported by grants from the Medizinisch-Wissenschaftlicher Fonds des Bürgermeisters der Bundeshauptstadt Wien, Jubiläumsfonds der österreichischen Nationalbank, österreichischen Gesellschaft für Chemotherapie, Theodor Körner Fonds zur Förderung von Wissenschaft und Kunst and the Kamillo Eisner Stiftung. Discussions with the Preclinical Therapeutic Models Group (PTMG) encouraged elaboration of this study.

### References

BAIOCCHI G, KAVANAGH JJ, TALPAZ M, WHARTON JT, GUTTERMAN JU AND KURZROCK R. (1991). Expression of macrophage colony stimulating factor and its receptor in gynecologic malignancies. *Cancer*, **67**, 990-996.

BAST JR RC, BOYER CM, JACOBS I, XU FJ, WU S, WIENER J, KOHLER M AND BERCHUCK A. (1993). Cell growth regulation in epithelial ovarian cancer. *Cancer*, **71**, 1597-1601.

BERCHUCK A, KOHLER MF, BOENTLE MB, RODRIGUEZ GC, WHITAKER RS AND BAST JR RC. (1993). Growth regulation and transformation of ovarian epithelium. *Cancer*, **71**, 545-551.

BUICK RN, PULLANO P AND TRENT JM. (1985). Comparative properties of five human ovarian adenocarcinoma cell lines. *Cancer Res.*, **45**, 3668-3676.

CHEN AR AND ROHRSCHNEIDER LR. (1993). Mechanism of differential inhibition of factor dependent cell proliferation by transforming growth factor-$\beta$1: selective uncoupling of FMS from MYC. *Blood*, **81**, 2539-2546.

EDELMAN AM, BLUMENTHAL DK AND KREBS EG. (1987). Protein serine/threonine kinases. *Ann. Rev. Biochem.*, **56**, 567-613.

FILMUS JE AND BUICK RN. (1985). Stability of c-K-ras amplification during progression in a patient with adenocarcinoma of the ovary. *Cancer Res.*, **46**, 4468-4472.

GRUNT TW, DITTRICH E, SOMAY C, WAGNER T AND DITTRICH C. (1991*a*) Separation of clonogenic and differentiated cell phenotypes of ovarian cancer cells (HOC-7) by discontinuous density gradient centrifugation. *Cancer Lett.*, **58**, 7-16.

GRUNT TW, SOMAY C, PAVELKA M, ELLINGER A, DITTRICH E AND DITTRICH C. (1991*b*). The effects of dimethyl sulfoxide and retinoic acid on the cell growth and the phenotype of ovarian cancer cells. *J. Cell Sci.*, **100**, 657-666.

GRUNT TW, SOMAY C, OELLER H, DITTRICH E AND DITTRICH C. (1992*a*). Comparative analysis of the effects of dimethyl sulfoxide and retinoic acid on the antigenic pattern of human ovarian adenocarcinoma cells. *J. Cell Sci.*, **103**, 501-509.

GRUNT TW, SOMAY C, ELLINGER A, PAVELKA M, DITTRICH E AND DITTRICH C. (1992*b*). The differential effects of N,N,-dimethylformamide and transforming growth factor-$\beta$1 on a human ovarian cancer cell line (HOC-7). *J. Cell Physiol.*, **151**, 13-22.

GRUNT TW, OELLER H, SOMAY C AND DITTRICH C. (1993*a*). Different propensity for spontaneous differentiation of cell clones isolated from the human ovarian surface epithelial cell line HOC-7. *Differentiation*, **53**, 45-50.

GRUNT TW, OELLER H, SOMAY C, DITTRICH E, FAZENY B, MANNHALTER C AND DITTRICH C. (1993*b*). Modulation of the immunophenotype of ovarian cancer cells by N,N-dimethylformamide and transforming growth factor $\beta$1. *J. Cell Physiol.*, **156**, 358-366.

HANN SR, THOMPSON CB AND EISENMAN RN. (1985). c-myc oncogene protein synthesis is independent of the cell cycle in human and avian cells. *Nature*, **314**, 366-369.

HENRIKSEN R, FUNA K, WILANDER E, BACKSTROM T, RIDDERHEIM M AND OBERG K. (1993). Expression and prognostic significance of platelet-derived growth factor and its receptors in epithelial ovarian neoplasms. *Cancer Res.*, **53**, 4550-4554.

KACINSKI BM, CARTER D, MITTAL K, YEE LD, SCATA KA, DONOFRIO L, CHAMBERS SK, WANG KI, YANG-FENG T, ROHRSCHNEIDER LR AND ROTHWELL VM. (1990). Ovarian adenocarcinomas express fms-complementary transcripts and fms antigen, often with coexpression of CSF-1. *Am. J. Pathol.*, **137**, 135-147.

KRUPITZA G, HULLA W, HARANT H, DITTRICH E, KALLAY E, HUBER H AND DITTRICH C. (1995). Retinoic acid induced death of ovarian carcinoma cells correlates with c-myc stimulation. *Int. J. Cancer*, (in press).

KURACHI H, MORISHIGE K, AMEMIYA K, ADACHI H, HIROTA K, MIYAKE A AND TANIZAWA O. (1991). Importance of transforming growth factor alpha/epidermal growth factor, receptor autocrine growth mechanism in an ovarian cancer cell line in vivo. *Cancer Res.*, **51**, 5956-5959.

LI BY, MOHNRAJ D, OLSON MC, MORADI M, TWIGGS L, CARSON LF AND RAMAKRISHNAN S. (1992). Human ovarian epithelial cancer cell cultures in vitro express both interleukin 1 alpha and beta genes. *Cancer Res.*, **52**, 2248-2252.

MALIK S AND BALKWILL FR. (1991). Epithelial ovarian cancer: a cytokine propelled disease? *Br. J. Cancer*, **64**, 617-620.

NAYLOR MS, STAMP GW, FOULKES WD, ECCLES D AND BALKWILL FR. (1993). Tumor necrosis factor and its receptors in human ovarian cancer. *J. Clin. Invest.*, **91**, 2194-2206.

RAO BR AND SLOTMAN BJ. (1991). Endocrine factors in common epithelial ovarian cancer. *Endocrinol Rev.* **12**, 14-26.

RESNITZKY D, YARDEN A, ZIPORI D AND KIMCHI A. (1986). Autocrine $\beta$-related interferon controls c-myc suppression and growth arrest during hematopoietic cell differentiation. *Cell*, **46**, 31-40.

SOMAY C, GRUNT TW, MANNHALTER C AND DITTRICH C. (1992). Relationship of myc protein expression to the phenotype and to the growth potential of HOC-7 ovarian cancer cells. *Br. J. Cancer*, **66**, 93-98.

SPOTTS GD AND HANN SR. (1990). Enhanced translation and increased turnover of c-myc proteins occur during differentiation of murine erythroleukemia cells. *Mol. Cell. Biol.*, **10**, 3952-3964.

SUZUKI M, OHWADA M, AIDA I, SATO I AND TAMADA T. (1994). Macrophage-colony stimulating factor enhances platelet recovery following cisplatin/carboplatin chemotherapy in ovarian cancer. *Gynecol. Oncol.*, **54**, 23-26.

WATSON JM, BEREK JS AND MARTINEZ-MAZA O. (1993). Growth inhibition of ovarian cancer cells induced by antsense IL-6 oligonucleotides. *Gynecol. Oncol.*, **49**, 8-15.

WIENER JR, BERCHUCK A AND BAST JR RC. (1992). Biology and therapy with biologic agents in gynecologic cancer. *Curr. Opin. Oncol.*, **4**, 946-954.

WU S, BOYER CM, WHITAKER RS, BERCHUCK A, WIENER JR, WEINBERG JB AND BAST JR RC. (1993). Tumor necrosis factor alpha as an autocrine and paracrine growth factor for ovarian cancer: monokine induction of tumor cell proliferation and tumor necrosis factor alpha expression. *Cancer Res.*, **53**, 1939-1944.

XU XX, TESSNER TG, ROCK CO AND JACKOWSKY S. (1993). Phosphatidylcholine hydrolysis and c-myc expression are in collaborating mitogenic pathways activated by colony stimulating factor 1. *Mol. Cell. Biol.*, **13**, 1522-1533.

YEE D, MORALES FR, HAMILTON TC AND VON HOFF DD. (1991). Expression of insulin-like growth factor I, its biding proteins and its receptor in ovarian cancer. *Cancer Res.*, **51**, 5107-5112.